# Association between the Oxidative Balance Score and Incident Chronic Kidney Disease in Adults

**DOI:** 10.3390/antiox12020335

**Published:** 2023-01-31

**Authors:** Da-Hye Son, Hye Sun Lee, So-Young Seol, Yong-Jae Lee, Jun-Hyuk Lee

**Affiliations:** 1 Department of Family Medicine, Gangnam Severance Hospital, Yonsei University College of Medicine, Seoul 06273, Republic of Korea; 2 Department of Integrative Medicine, Yonsei University Graduate School, Seoul 03722, Republic of Korea; 3 Biostatistics Collaboration Unit, Department of Research Affairs, Yonsei University College of Medicines, Seoul 06273, Republic of Korea; 4 Department of Family Medicine, Nowon Eulji Medical Center, Eulji University School of Medicine, Seoul 01830, Republic of Korea; 5 Department of Medicine, Graduate School of Hanyang University, Seoul 04763, Republic of Korea

**Keywords:** oxidative balance score, chronic kidney disease, incidence, prospective cohort

## Abstract

Oxidative stress is a novel risk factor for chronic kidney disease (CKD). The oxidative balance score (OBS) was developed to represent the overall oxidative balance based on dietary and lifestyle pro-oxidant and antioxidant components. The aim of this study is to verify the relationship between the OBS and the incidence of CKD. Data from 5795 participants without CKD at the baseline survey of the Korean Genome and Epidemiology Study were analyzed. Participants were classified into sex-specific OBS tertiles. During the mean follow-up period of 13.6 years, 286 men and 382 women newly developed CKD. The Cox proportional hazard spline curve revealed an inverse dose–response association between the OBS and incident CKD in both men and women. Multiple Cox proportional hazard regression analysis revealed that the adjusted hazard ratios (95% confidence intervals) for sex-specific highest (T3) and middle (T2) OBS tertile groups were 0.80 (0.59–1.08) and 0.70 (0.51–0.95), respectively, in men and 0.76 (0.59–0.98) and 0.73 (0.55–0.96), respectively, in women, with the sex-specific lowest OBS tertile group (T1) as the reference. These results suggest that a healthy diet and lifestyle that increases the OBS may help prevent CKD in both men and women.

## 1. Introduction

Chronic kidney disease (CKD) is a growing health issue worldwide that is associated with a high economic burden, including clinic visits, dialysis, and medical costs [1,2]. The global prevalence of CKD was estimated to be approximately 700 million in 2017, with a 29.3% increase since 1990 [3]. CKD is the 12th leading cause of death, with 1.2 million deaths globally [3]. Therefore, to reduce the health burden caused by CKD, it is important to develop effective screening tools to identify risk factors. Smoking, obesity, hypertension, and diabetes mellitus are well-known modifiable risk factors for CKD [4].

Recent studies have shown that oxidative stress, defined as an imbalance between the production of reactive oxygen species and antioxidant defense capacity, is a novel risk factor for CKD [5,6,7]. Oxidative stress causes damage to macromolecules and DNAs and abnormal cell signaling [8]. As the kidney is a mitochondria-rich organ with a high energy demand, it is more vulnerable to oxidative stress [9]. In addition, oxidative stress can progress to CKD and contribute to complications, including cardiovascular and neurological complications [10,11]. Several studies have demonstrated that pro-oxidant biomarker levels are elevated and antioxidant biomarker levels are reduced in patients with CKD [12,13,14,15]. However, pro- or antioxidant biomarkers have limitations in indicating the overall oxidative stress burden. Thus, the oxidative balance score (OBS) was developed to represent the overall oxidative balance based on dietary and lifestyle pro- and antioxidant components [16]. Since the OBS was first defined by Van Hoydonck et al. [17] in 2002, more than 20 modified versions of the OBS have been published by selecting different factors or applying different scoring systems to improve the quality of this particular scoring system [18]. In the OBS, antioxidant components contribute positively, whereas pro-oxidant components contribute negatively; thus, lower OBSs indicate a higher pro-oxidant burden.

To date, only one cohort study in the United States has shown an inverse association between the OBS and new-onset CKD [19]. However, there are racial/ethnic differences in the development and progression of CKD [20,21]. Therefore, the aim of this study was to evaluate the association between the OBS and the risk of new-onset CKD in East Asian middle-aged and older adults, using community-based, large-scale Korean cohort data.

## 2. Materials and Methods

### 2.1. Study Population

Data from the Korean Genome and Epidemiology Study (KoGES)_Ansan and Ansung cohorts were used. The KoGES_Ansan and Ansung cohorts were established to verify the genetic and epidemiologic risk factors for non-communicable diseases. The detailed study design and procedures have been described in a previous study [22]. A total of 10,030 community-dwelling adults aged 40–69 years participated in the baseline survey (2001–2002) and were followed up biennially to the eighth follow-up year (2017–2018).

**Figure 1:**
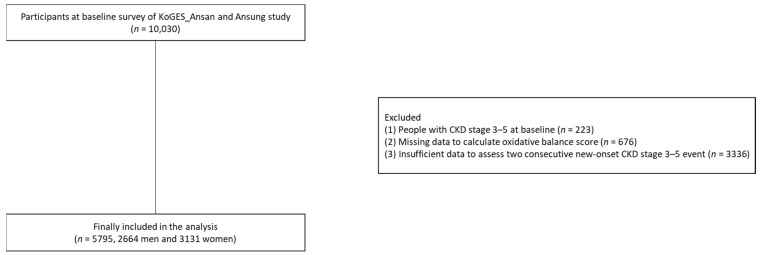
Flowchart of the study population. Abbreviations: KoGES, Korean Genome and Epidemiology Study; CKD, chronic kidney disease.

Among 10,030 participants at baseline, we excluded patients with (1) CKD stages 3–5 at baseline (*n* = 223), (2) insufficient data to calculate the OBS (*n* = 676), and (3) insufficient data to assess two consecutive events of new-onset CKD stages 3–5 during follow-up (*n* = 3336). Finally, 5795 participants were included in the analysis (Figure 1).

Informed consent was obtained from all participants. In accordance with the 1964 Declaration of Helsinki and its later amendments, the study protocol was approved by the institutional review board (IRB) of Nowon Eulji Medical Center (IRB number: 2021-03-009).

### 2.2. Assessment of OBS

The OBS was totalized by calculating eight pro-oxidant and seven antioxidant factors selected based on previous studies [23,24,25,26]. Table 1 describes the OBS scheme [27]. Pro-oxidant factors included saturated fatty acid (SFA), omega-6 polyunsaturated fatty acid (PUFA), copper intake, total iron intake, cigarette smoking status, alcohol drinking status, obesity status, and abdominal obesity status. The sex-specific tertile values of SFA, omega-6 PUFA, total iron intake, and copper intake were scored corresponding to the lowest (score 2), middle (score 1), and highest (score 0) tertiles, respectively. Current smokers, former smokers, and never smokers were assigned scores of 0, 1, and 2 points, respectively. Regarding alcohol drinking status, heavy drinkers (≥30 g/day in men, ≥20 g/day in women), mild-to-moderate drinkers (1–29 g/day in men, 1–19 g/day in women), and non-drinkers were assigned 0, 1, and 2 points, respectively. As for obesity, overweight, and normal weight, the scores were 0, 1, and 2, respectively. For abdominal obesity, 0 points were assigned for abdominal obesity and 1 point for a normal abdomen. Antioxidant factors included the intake of omega-3 PUFA, vitamin C, vitamin E, selenium, beta-carotene, and zinc, as well as physical activity. The sex-specific tertile values of each variable, omega-3 PUFA, vitamin C, vitamin E, selenium, beta-carotene, and zinc intake were scored corresponding to the lowest (score 0), middle (score 1), and highest tertiles (score 2). Low-intensity physical activity received 0 points, moderate-intensity physical activity received 1 point, and high-intensity physical activity received 2 points. The sum of the OBS ranged from 0 to 29 points. Using the OBS, we divided the individuals into tertile groups in a sex-specific manner.

### 2.3. Definition of CKD

The primary endpoint was new-onset CKD defined as two newly developed consecutive events of an estimated glomerular filtration rate (eGFR) <60 mL/min/1.73 m^2^. The eGFR was calculated using the CKD Epidemiology Collaboration (CKD-EPI) equation [28].

### 2.4. Covariates

Variables from the baseline survey were used as covariates in the analysis. Each participant’s height (m) and body weight (kg) were measured to the nearest 0.001 m and 0.1 kg, respectively. A body mass index (BMI) ≥25 kg/m^2^ indicated obesity, and BMI ≥ 23 and <25 kg/m^2^ indicated overweight based on the definition of the 2018 Korean Society for the Study of Obesity [29]. Systolic blood pressure (SBP) and diastolic blood pressure (DBP) were measured, then we calculated mean blood pressure (MBP). Hypertension was defined as SBP ≥ 140 mmHg, DBP ≥ 90 mmHg, or treatment with antihypertensive medications [30].

Blood samples were collected after ≥8 h of fasting and were analyzed with a chemical analyzer. Fasting plasma glucose, creatinine, total cholesterol, high-density lipoprotein cholesterol, triglyceride, and C-reactive protein levels were enzymatically analyzed (Hitachi 7600, Hitachi, Tokyo, Japan in August 2002 and ADVIA 1650, Siemens, Tarrytown, NY, USA from September 2002). Low-density lipoprotein cholesterol levels were calculated using the Friedewald equation for serum triglyceride levels <400 mg/dL [31].

A 103-item food frequency questionnaire (FFQ) was used to estimate the amount of macro-/micronutrient consumed by each participant. Well-trained dietitians conducted in-person interviews with participants for dietary surveillance. The total energy (kcal/day), protein (g/day), SFA (g/day), omega-6-PUFA (g/day), total iron (mg/day), copper (μg/day), omega-3 PUFA (g/day), vitamin C (mg/day), vitamin E (mg/day), selenium (μg/day), beta-carotene (μg/day), and zinc (mg/day) intake of each participant were calculated from the FFQ using a computer-aided nutritional analysis program (CAN-pro version 5.0, The Korean Nutrition Society, Seoul, Korea). The protein-to-total energy intake (%) was calculated as 100 × 4 (kcal/g) × protein intake (g/day)/total energy intake (kcal/day).

Participants also completed a self-report questionnaire regarding smoking, alcohol consumption, physical activity, level of education, and monthly household income. The status of smoker was classified as never smoker, former smoker, or current smoker. Those who had never smoked or had smoked less than 100 cigarettes in their lifetime were considered never smokers. Former smokers were defined as adults who had quit smoking at the time of the interview and had smoked at least 100 cigarettes in their lifetime. Current smokers were defined as adults who currently smoked and had smoked at least 100 cigarettes in their lifetime. Alcohol consumption per day (g/day) was calculated by multiplying the amount of alcohol consumption per time (glasses/time) by the alcohol consumption frequency (times/month) × 10 (g/per glass of drink)/30 (days/month). Physical activity levels were measured by asking people how many metabolic equivalent of tasks (MET) they perform on a daily basis (Met-h/day) using the International Physical Activity Questionnaire [32]. Low (<7.5 MET-h/day), moderate (7.5–30 MET-h/day), and high (>30 MET-h/day) were the categories categorized by physical activity [33]. The education level was classified as (1) elementary school or middle school, (2) high school, or (3) college or university. The monthly household income was divided into three groups: (1) <1 million Korean Won (KRW), (2) 1–2 million KRW, and (3) >2 million KRW.

### 2.5. Statistical Analysis

All data were analyzed separately according to sex. All data are presented as means ± standard deviations for continuous variables and numbers (percentages, %) for categorical variables. For continuous variables, analysis of variance was used to compare differences between sex-specific OBS tertile groups. For categorical variables, the chi-square test was used.

Cox proportional hazard spline curves were drawn to determine the dose–response relationship between the OBS and incident CKD in men and women. We used Kaplan–Meier curves with log-rank tests to determine the cumulative incidence rate of CKD according to the sex-specific OBS tertile groups. Multiple Cox proportional hazard regression analysis was performed to estimate hazard ratios (HRs) with 95% confidence intervals (CIs) for incident CKD in the sex-specific highest (T3) and middle (T2) OBS tertile groups compared with the sex-specific lowest (T1) OBS tertile group. The HR with 95% CI for incident CKD per increment of OBS was also verified. In the adjusted model, age, education level, monthly household income, ratio of protein intake to total energy intake, MBP, serum total cholesterol level, and serum C-reactive protein level were included as confounding variables.

We used SAS statistical software (version 9.4; SAS Institute Inc., Cary, NC, USA) and R software (version 4.1.1; R Foundation for Statistical Computing, Vienna, Austria) to perform the statistical analyses. Statistical significance was set at a *p* value < 0.05.

## 3. Results

### 3.1. Characteristics of the Study Population

Table 2 presents the baseline characteristics of the study population across total and sex-specific OBS tertile groups. The T3 group showed the highest proportion of participants with the highest education level, household income in total population and percentage of women. The mean values of MBP, glucose, insulin, total cholesterol, and triglyceride levels in the total population and in both men and women were highest in the T1 group. The mean values of age were highest in the T1 group among total study population and women. The T3 group exhibited the highest mean values for energy intake and protein/total energy intake in total population and both sex. The values for the high-density lipoprotein cholesterol level and eGFR were highest in the T3 group across total group and women.

### 3.2. Individual Components of the OBS According to Sex-Specific OBS Tertile

Table 3 displays the clinical characteristics of the individual OBS components according to the OBS tertiles. The mean intake of SFA, omega-6 PUFA, total iron, total copper, omega-3 PUFA, vitamin C, vitamin E, beta-carotene, and zinc intake increased across the total, male and female tertiles of OBS. Participants with obesity, current smokers, heavy drinkers, and low-intensity physical activity were more prevalent among the T1 group for both sexes and total study population.

### 3.3. Relationship between Oxidative Balance Score and Incident Chronic Kidney Disease

**Figure 2:**
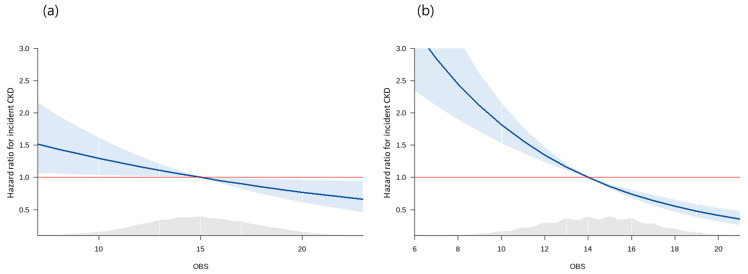
Spline curves for the Cox proportional hazard model of incident CKD according to OBS in (**a**) men and (**b**) women. Abbreviations: CKD, chronic kidney disease; OBS, oxidative balance score.

A total of 286 men and 382 women developed new-onset CKD during a mean follow-up period of 13.6 years. Cox proportional hazard spline curves showed that incident CKD was inversely associated with the OBS in a dose-dependent manner (Figure 2).

Table 4 presents the independent association between new-onset CKD and the OBS using HRs and 95% CIs. Compared with T1, the HRs (95% CIs) for new-onset CKD in the T2 and T3 groups were 0.76 (0.63–0.91) and 0.57 (0.48–0.69) in the overall population, respectively, and 0.94 (0.71–1.25) and 0.74 (0.56–0.98), respectively, in men and 0.66 (0.53–0.84) and 0.48 (0.37–0.62), respectively, in women. After adjusting for age, education level, monthly household income, protein/total energy intake, MBP, serum total cholesterol level, and C-reactive protein level, the adjusted HRs (95% CIs) for T2 and T3 were 0.77 (0.63–0.93) and 0.71 (0.58–0.87), respectively, in overall population and 0.80 (0.59–1.08) and 0.70 (0.51–0.95), respectively, in men and 0.76 (0.59–0.98) and 0.73 (0.55–0.96), respectively, in women. The adjusted HRs and 95% CIs for incident CKD for each increment of 1 in the OBS were 0.94 (0.91–0.97) in overall population, 0.95 (0.90–0.99) in men and 0.93 (0.89–0.98) in women.

**Figure 3:**
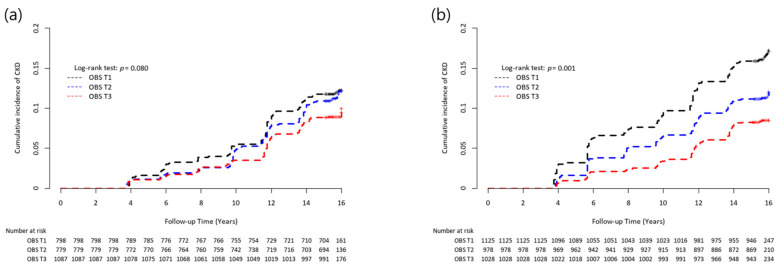
Inverse Kaplan–Meier curves of incident CKD according to OBS tertile groups in (**a**) men and (**b**) women. Abbreviations: CKD, chronic kidney disease, OBS, oxidative balance score.

The Kaplan–Meier curve with the log-rank test showed the highest risk of cumulative CKD incidence in the T1 group, followed by the T2 and T3 groups in both men and women (Figure 3).

## 4. Discussion

This large-scale prospective study is one of the few to examine the association between the OBS and the risk of CKD. Incident CKD occurred in 10.7% of men and 12.2% of women during the follow-up period, showing a high prevalence in women. Although gender difference in CKD prevalence vary across the countries, the results of this study are similar to those in a meta-analysis of previous studies comparing CKD prevalence in Asian countries, where the prevalence was higher in women but the severity and mortality rates were higher in men [34]. Additionally, the results of this study suggested that a lower OBS at baseline was associated with an increased risk of CKD during the 16.8-year follow-up in both men and women. After adjustment, the OBS was still inversely related to the risk of new-onset CKD. The adjusted HRs and 95% Cis for incident CKD for each increment of 1 in the OBS were 0.95 (0.90–0.99) in men and 0.93 (0.89–0.98) in women, showing lower HRs in women. This discrepancy suggests that oxidative stress is physiologically different in men and women. There has been some evidence that females have a greater antioxidant capacity than males [35], possibly owing to sex differences in antioxidant enzyme expression and activity [36]. Particularly, estradiol decreases NADPH oxidase activity and expression, generating reactive oxygen species, whereas it enhances antioxidant enzymes such as superoxide dismutase and glutathione peroxidase [37]. Furthermore, estrogen scavenges free radicals via its phenolic hydroxyl group [38]. In this regard, the results of this study suggest that men without the antioxidant potential of estradiol need to follow more anti-oxidant lifestyles and diets to reduce the risk of CKD.

The mean values of SFA, omega-6, total iron, and copper intake showed opposite trends compared to other pro-oxidant components. This discrepancy may be due to the effect of total energy intake, which tends to increase with increasing OBS tertiles. Additionally, because OBS is the sum of its various components, each component may show an independent trend.

Our findings are in line with those of previous epidemiological studies that have shown a negative link between oxidative stress and CKD progression. The levels of various oxidative stress markers, such as plasma 8-isoprostane, malondialdehyde, and oxidized serum albumin, seem to increase as CKD progresses and are inversely associated with the glomerular filtration rate [5,39,40]. Another cross-sectional study conducted in 116 patients with CKD also showed similar results using plasma levels of 15-F_2t_-isoprostane, which is a highly accurate oxidative stress marker in vivo in humans [41]. In contrast, the levels of various antioxidant markers, such as glutathione peroxidase, plasma thiol, superoxide dismutase, and catalase, seemed to be reduced in CKD patients [5,42,43]. Because these biomarkers are not widely used in clinical practice and there is no single marker that reflects the overall oxidative stress burden, the OBS used in this study can be applied in clinical practice, as it provides a cost-effective method to evaluate the overall oxidative stress balance using only dietary and lifestyle questionnaires without blood or urine tests.

Ilori et al. [19] reported that higher OBS quartiles were significantly associated with a lower risk of CKD, whereas OBS quartiles were not significantly associated with albuminuria and incident ESRD. Although this study is the first to show a negative relationship between OBS and the risk of new-onset CKD, most of the study population included Caucasians. As dietary and lifestyle factors constituting OBS are highly influenced by race and culture [20,21], similar results from our study support previous evidence that a healthy diet and lifestyle, which lower oxidative stress, reduce the risk of incident CKD in Asian populations.

In a meta-analysis of 103 observational studies, modifiable lifestyle factors for preventing CKD included a high intake of fruits, vegetables, and potassium-rich food; higher physical activity levels; moderate alcohol consumption; low intake of sodium; and smoking cessation [44]. Most of these factors were included in the OBS used in this study. Although a meta-analysis described these factors as contributing to the prevention of CKD by reducing the risk of obesity and lowering blood pressure, reducing the oxidative stress burden is also thought to be relevant. In this respect, we believe that OBS can be a useful tool for educating the general population on healthy diet and lifestyle patterns to prevent CKD, in addition to its value as a screening tool to predict the risk of CKD.

Although the etiology of CKD is complex, the most likely explanation for the relationship between oxidative stress and incident CKD is endothelial dysfunction and chronic inflammation. High levels of oxidative stress and chronic inflammation decrease nitric oxide bioavailability, leading to endothelial dysfunction [45]. Endothelial dysfunction contributes to impaired renal function through anatomical alterations [46]. In addition, a recent study demonstrated that oxidative stress induces autophagy and apoptosis in podocytes, which maintain the glomerular filtration barrier [47].

This study has some limitations. First, because all the components used in the OBS were equally weighted, they may not have appropriately represented the actual biological contributions. However, studies indicated that there was no significant difference between weighted and unweighted OBS regarding the association with the risk of colorectal adenoma and prostate cancer [24,48]. Second, antioxidants have a threshold effect above which they can show toxic pro-oxidant activity at high doses, but OBS did not consider this and was defined under the assumption that the properties of all components linearly affect oxidative stress [49]. Third, we calculated each nutritional component based on the FFQ; therefore, recall and selection bias may exist. In addition, we could not analyze the changes in OBSs over time because we only had baseline FFQ data. Finally, the OBS used in this study does not include intake of medication, such as NSAIDs and aspirin that may affect oxidative stress levels due to lack of data. Despite these limitations, we believe that our finding of an inverse relationship between the OBS and incident CKD in a prospective cohort study is clinically valuable.

## 5. Conclusions

In this prospective cohort study, a higher OBS, which indicates a predominance of antioxidant exposure, was associated with a lower risk of incident CKD after adjustments for confounding factors. This study suggests that a healthy diet and lifestyle that increases OBS may help prevent CKD in both men and women.

## Figures and Tables

**Table 1:** Oxidative balance score assignment scheme.

OBS Components	Assignment Scheme *
1. Saturated fatty acid [P]	0 = high (3rd tertile), 1 = intermediate (2nd tertile), 2 = low (1st tertile)
2. Omega-6 PUFA intake [P]	0 = high (3rd tertile), 1 = intermediate (2nd tertile), 2 = low (1st tertile)
3. Total iron intake [P]	0 = high (3rd tertile), 1 = intermediate (2nd tertile), 2 = low (1st tertile)
4. Copper intake [P]	0 = high (3rd tertile), 1 = intermediate (2nd tertile), 2 = low (1st tertile)
5. Smoking status [P]	0 = current smoker, 1 = former smoker, 2 = never smoker
6. Alcohol drinking status [P]	0 = heavy drinker, 1 = mild-to-moderate drinker, 2 = non-drinker
7. Overweight/obese [P]	0 = obese, 1 = overweight, 2= normal
8. Abdominal obesity [P]	0 = abdominal obesity, 1 = normal
9. Omega-3 PUFA intake [A]	0 = low (1st tertile), 1 = intermediate (2nd tertile), 2 = high (3rd tertile)
10. Vitamin C intake [A]	0 = low (1st tertile), 1 = intermediate (2nd tertile), 2 = high (3rd tertile)
11. Vitamin E intake [A]	0 = low (1st tertile), 1 = intermediate (2nd tertile), 2 = high (3rd tertile)
12. Selenium intake [A]	0 = low (1st tertile), 1 = intermediate (2nd tertile), 2 = high (3rd tertile)
13. Total beta-carotene intake [A]	0 = low (1st tertile), 1 = intermediate (2nd tertile), 2 = high (3rd tertile)
14. Zinc intake [A]	0 = low (1st tertile), 1 = intermediate (2nd tertile), 2 = high (3rd tertile)
15. Physical activity [A]	0 = low intensity, 1 = moderate intensity, 2 = high intensity

* Low, intermediate, and high categories correspond to sex-specific tertile values among participants in the KoGES at the baseline survey. Abbreviations: OBS, oxidative balance score; PUFA, polyunsaturated fatty acid. (Reprinted with permission of authors [27].)

**Table 2:** Baseline characteristics of the study population.

	Oxidative Balance Score											
	Total				Men				Women			
Variables	T1 (*n* = 1923)	T2 (*n* = 1757)	T3 (*n* = 2115)	p *	T1 (*n* = 798)	T2 (*n* = 779)	T3 (*n* = 1087)	p *	T1 (*n* = 1125)	T2 (*n* = 978)	T3 (*n* = 1028)	p *
Men, *n* (%)	798 (41.5%)	779 (44.3%)	1087 (51.4%)	<0.001	N/A	N/A	N/A		N/A	N/A	N/A	
Age, years	52.4 ± 8.4	51.8 ± 8.5	50.3 ± 8.2	<0.001	50.5 ± 7.7	51.5 ± 8.4	50.8 ± 8.2	0.593	53.8 ± 8.6	52.1 ± 8.7	49.9 ± 8.1	<0.001
MBP, mmHg	98.0 ± 13.2	96.7 ± 12.7	94.0 ± 12.6	<0.001	98.4 ± 12.2	98.1 ± 11.9	96.2 ± 12.3	<0.001	97.6 ± 13.8	95.6 ± 13.3	91.7 ± 12.6	<0.001
Glucose, mg/dL	87.3 ± 18.8	86.6 ± 18.9	85.7 ± 20.6	0.008	90.1 ± 20.8	89.0 ± 18.6	88.4 ± 23.2	0.089	85.3 ± 17.0	84.6 ± 19.0	82.8 ± 16.8	0.001
Insulin, µU/mL	8.2 ± 5.5	7.7 ± 4.7	7.2 ± 4.0	<0.001	7.4 ± 4.5	7.2 ± 4.0	6.8 ± 3.6	<0.001	8.7 ± 6.1	8.1 ± 5.1	7.6 ± 4.3	<0.001
Total cholesterol, mg/dL	194.7 ± 34.5	191.0 ± 35.7	187.8 ± 33.5	<0.001	195.4 ± 34.9	193.4 ± 36.2	190.3 ± 34.3	0.002	194.1 ± 34.2	189.1 ± 35.2	185.0 ± 32.5	<0.001
Triglyceride, mg/dL	176.6 ± 108.5	160.3 ± 102.7	147.8 ± 94.8	<0.001	196.3 ± 121.9	180.2 ± 123.0	161.3 ± 101.9	<0.001	162.6 ± 95.4	144.3 ± 79.6	133.5 ± 84.4	<0.001
HDL cholesterol, mg/dL	43.3 ± 9.3	44.7 ± 10.2	45.1 ± 9.9	<0.001	42.3 ± 9.2	43.3 ± 9.7	43.9 ± 10.0	0.001	44.0 ± 9.2	45.8 ± 10.4	46.4 ± 9.7	<0.001
CRP, mg/dL	0.2 ± 0.4	0.3 ± 0.8	0.2 ± 0.4	0.016	0.2 ± 0.3	0.3 ± 0.8	0.2 ± 0.3	0.157	0.2 ± 0.5	0.2 ± 0.8	0.2 ± 0.4	0.041
eGFR, mL/min/1.73 m^2^	91.8 ± 13.2	92.8 ± 13.2	94.2 ± 12.8	<0.001	91.2 ± 13.4	91.5 ± 13.4	91.8 ± 13.0	0.321	92.2 ± 13.0	93.9 ± 12.9	96.6 ± 12.2	<0.001
Education level, *n* (%)				<0.001				0.928				<0.001
Elementary/middle school	1165 (60.9%)	951 (54.5%)	1018 (48.3%)		312 (39.1%)	303 (39.1%)	437 (40.4%)		853 (76.5%)	648 (66.7%)	581 (56.7%)	
High school	529 (27.7%)	559 (32.0%)	747 (35.5%)		307 (38.5%)	303 (39.1%)	402 (37.1%)		222 (19.9%)	256 (26.4%)	345 (33.7%)	
College/university	219 (11.4%)	236 (13.5%)	342 (16.2%)		179 (22.4%)	169 (21.8%)	244 (22.5%)		40 (3.6%)	67 (6.9%)	98 (9.6%)	
Household income, *n* (%)				<0.001				0.159				<0.001
<100 million Korean Won	706 (37.2%)	603 (34.7%)	588 (28.1%)		187 (23.6%)	204 (26.3%)	265 (24.5%)		519 (46.9%)	399 (41.5%)	323 (31.9%)	
100–200 million Korean Won	545 (28.7%)	499 (28.7%)	633 (30.2%)		242 (30.6%)	212 (27.4%)	352 (32.5%)		303 (27.4%)	287 (29.8%)	281 (27.8%)	
>200 million Korean Won	647 (34.1%)	635 (36.6%)	872 (41.7%)		363 (45.8%)	359 (46.3%)	465 (43.0%)		284 (25.7%)	276 (28.7%)	407 (40.3%)	
Total energy intake, kcal/day	1820.0 ± 528.8	2134.7 ± 755.2	2519.8 ± 868.2	<0.001	1926.4 ± 507.4	2209.1 ± 762.0	2556.4 ± 769.6	<0.001	1744.5 ± 531.0	2075.4 ± 744.8	2481.2 ± 960.4	<0.001
^†^ Protein/total energy intake, %	12.7 ± 2.0	13.3 ± 2.3	14.2 ± 2.4	<0.001	13.0 ± 2.0	13.5 ± 2.3	14.1 ± 2.3	<0.001	12.4 ± 2.0	13.2 ± 2.2	14.3 ± 2.5	<0.001

* p value for the comparison of the baseline characteristics among sex-specific tertile groups of oxidative balance score at the baseline survey. ^†^ Protein/total energy intake was calculated as 100 × 4 (kcal/g) × protein intake (g/day)/total energy intake (kcal/day). Statistical significance was set at *p* < 0.05. Abbreviations: MBP, mean blood pressure; HDL, high-density lipoprotein; CRP, C-reactive protein; eGFR, estimated glomerular filtration rate.

**Table 3:** Individual components of the score according to oxidative balance score tertiles.

	Oxidative Balance Score											
	Total				Men				Women			
Variables	T1 (*n* = 1923)	T2 (*n* = 1757)	T3 (*n* = 2115)	p *	T1 (*n* = 798)	T2 (*n* = 779)	T3 (*n* = 1087)	p *	T1 (*n* = 1125)	T2 (*n* = 978)	T3 (*n* = 1028)	p *
Saturated fatty acid, g/day	8.3 ± 4.4	10.5 ± 6.0	13.3 ± 8.4	<0.001	8.7 ± 4.0	10.6 ± 6.0	12.9 ± 7.4	<0.001	7.9 ± 4.6	10.4 ± 6.0	13.8 ± 9.3	<0.001
omega-6 PUFA intake, g/day	7.7 ± 4.2	8.8 ± 5.7	9.3 ± 5.4	<0.001	7.6 ± 3.8	8.8 ± 5.6	8.9 ± 5.0	<0.001	7.8 ± 4.4	8.8 ± 5.8	9.7 ± 5.9	<0.001
Total iron intake, mg/day	15.3 ± 6.1	19.0 ± 8.6	23.8 ± 11.3	<0.001	16.2 ± 6.2	19.2 ± 8.6	23.4 ± 10.0	<0.001	14.7 ± 6.1	18.8 ± 8.5	24.3 ± 12.5	<0.001
Copper intake, μg/day	1082.0 ± 821.5	1288.9 ± 892.7	1504.6 ± 916.4	<0.001	1168.1 ± 856.4	1294.4 ± 926.5	1398.1 ± 840.4	<0.001	1020.9 ± 790.5	1284.6 ± 865.3	1617.3 ± 978.3	<0.001
Smoking status, *n* (%)				<0.001				<0.001				<0.001
Current smoker	520 (27.0%)	390 (22.2%)	366 (17.3%)		466 (58.4%)	374 (48.0%)	363 (33.4%)		54 (4.8%)	16 (1.6%)	3 (0.3%)	
Former smoker	259 (13.5%)	257 (14.6%)	353 (16.7%)		242 (30.3%)	250 (32.1%)	350 (32.2%)		17 (1.5%)	7 (0.7%)	3 (0.3%)	
Never smoker	1144 (59.5%)	1110 (63.2%)	1396 (66.0%)		90 (11.3%)	155 (19.9%)	374 (34.4%)		1054 (93.7%)	955 (97.6%)	1022 (99.4%)	
Drinking status, *n* (%)				<0.001				<0.001				<0.001
Heavy drinker	259 (13.5%)	172 (9.8%)	127 (6.0%)		229 (28.7%)	162 (20.8%)	121 (11.1%)		30 (2.7%)	10 (1.0%)	6 (0.6%)	
Mild-to-moderate drinker	790 (41.1%)	654 (37.2%)	738 (34.9%)		448 (56.1%)	417 (53.5%)	547 (50.3%)		342 (30.4%)	237 (24.2%)	191 (18.6%)	
Non-drinker	874 (45.4%)	931 (53.0%)	1250 (59.1%)		121 (15.2%)	200 (25.7%)	419 (38.5%)		753 (66.9%)	731 (74.7%)	831 (80.8%)	
Obesity status, *n* (%)				<0.001				<0.001				<0.001
Obese	1230 (64.0%)	794 (45.2%)	516 (24.4%)		498 (62.4%)	346 (44.4%)	278 (25.6%)		732 (65.1%)	448 (45.8%)	238 (23.2%)	
Overweight	452 (23.5%)	443 (25.2%)	632 (29.9%)		176 (22.1%)	198 (25.4%)	319 (29.3%)		276 (24.5%)	245 (25.1%)	313 (30.4%)	
Normal weight	241 (12.5%)	520 (29.6%)	967 (45.7%)		124 (15.5%)	235 (30.2%)	490 (45.1%)		117 (10.4%)	285 (29.1%)	477 (46.4%)	
Abdominal obesity, *n* (%)	909 (47.3%)	533 (30.3%)	292 (13.8%)	<0.001	274 (34.3%)	190 (24.4%)	113 (10.4%)	<0.001	635 (56.4%)	343 (35.1%)	179 (17.4%)	<0.001
omega-3 PUFA intake, g/day	1.1 ± 0.7	1.3 ± 0.9	1.5 ± 0.9	<0.001	1.1 ± 0.6	1.3 ± 0.9	1.5 ± 0.9	<0.001	1.1 ± 0.7	1.3 ± 0.9	1.6 ± 1.0	<0.001
Vitamin C intake, mg/day	79.5 ± 59.3	123.7 ± 102.4	182.1 ± 133.8	<0.001	74.2 ± 42.4	109.1 ± 83.8	158.4 ± 113.6	<0.001	83.2 ± 68.6	135.3 ± 113.7	207.1 ± 148.2	<0.001
Vitamin E intake, mg/day	9.7 ± 4.3	13.6 ± 6.8	18.3 ± 9.1	<0.001	10.5 ± 4.2	13.7 ± 6.7	17.6 ± 7.7	<0.001	9.1 ± 4.3	13.5 ± 7.0	19.0 ± 10.4	<0.001
Selenium intake, μg/day	32.3 ± 19.4	47.2 ± 28.9	66.3 ± 41.6	<0.001	37.1 ± 20.2	49.5 ± 30.8	65.7 ± 34.9	<0.001	28.9 ± 18.0	45.3 ± 27.1	66.8 ± 47.8	<0.001
Beta-carotene intake, μg/day	2074.7 ± 1488.3	3385.7 ± 2861.2	5062.3 ± 4270.2	<0.001	2288.7 ± 1756.2	3508.6 ± 3073.5	4866.7 ± 3861.9	<0.001	1922.9 ± 1242.9	3287.8 ± 2677.6	5269.2 ± 4656.2	<0.001
Zinc intake, mg/day	10.5 ± 3.4	12.4 ± 4.7	14.8 ± 5.7	<0.001	10.7 ± 3.1	12.5 ± 4.7	14.7 ± 5.1	<0.001	10.3 ± 3.5	12.3 ± 4.7	15.0 ± 6.3	<0.001
Physical activity, *n* (%)				<0.001				<0.001				<0.001
Low (<7.5 METs-hr/wk)	230 (12.0%)	103 (5.9%)	78 (3.7%)		89 (11.2%)	28 (3.6%)	36 (3.3%)		141 (12.5%)	75 (7.7%)	42 (4.1%)	
Moderate (7.5–30 METs-hr/wk)	1198 (62.3%)	1098 (62.5%)	1193 (56.4%)		527 (66.0%)	485 (62.3%)	590 (54.3%)		671 (59.6%)	613 (62.7%)	603 (58.7%)	
High (>30 METs-hr/wk)	495 (25.7%)	556 (31.6%)	844 (39.9%)		182 (22.8%)	266 (34.1%)	461 (42.4%)		313 (27.8%)	290 (29.7%)	383 (37.3%)	

* p value for the comparison of the baseline characteristics among sex-specific tertile groups of oxidative balance score at baseline survey. Statistical significance was set at *p* < 0.05. Abbreviations: PUFA, polyunsaturated fatty acid.

**Table 4:** Cox proportional hazard regression analysis showing the relationship of the oxidative balance score with incident chronic kidney disease.

Oxidative Balance Score Tertile	Numbers, *n*	New-Onset Cases, *n*	Follow-Up Period, Person–Year	Incidence Rate Per 1000 Person Years	Unadjusted	Adjusted
					HR (95% CI)	HR (95% CI)
Total						
Continuous (per 1 increment)					0.90 (0.87–0.93)	0.94 (0.91–0.97)
T1	1923	288	28,539.5	10.09	1 (reference)	1 (reference)
T2	1757	203	26,507.1	7.66	0.76 (0.63–0.91)	0.77 (0.63–0.93)
T3	2115	187	32,302.5	5.79	0.57 (0.48–0.69)	0.71 (0.58–0.87)
*p* for trend					<0.001	<0.001
Men						
Continuous (per 1 increment)					0.95 (0.91–0.99)	0.95 (0.90–0.99)
T1	798	97	12,012.2	8.08	1 (reference)	1 (reference)
T2	779	90	11,802.2	7.63	0.94 (0.71–1.25)	0.80 (0.59–1.08)
T3	1087	99	16,552.1	5.98	0.74 (0.56–0.98)	0.70 (0.51–0.95)
*p* for trend					0.031	0.022
Women						
Continuous (per 1 increment)					0.86 (0.83–0.90)	0.93 (0.89–0.98)
T1	1125	191	16,527.3	11.56	1 (reference)	1 (reference)
T2	978	113	14,704.9	7.68	0.66 (0.53–0.84)	0.76 (0.59–0.98)
T3	1028	88	15,750.4	5.59	0.48 (0.37–0.62)	0.73 (0.55–0.96)
*p* for trend					<0.001	0.015

Adjusted for age, education level, monthly household income, protein/total energy intake (%), mean blood pressure, serum total cholesterol level, and serum C-reactive protein level. Abbreviations: HR, hazard ratio; CI, confidence interval.

## Data Availability

The dataset used in this study can be provided after a review and evaluation of the research plan by the Korea Centers for Disease Control and Prevention (http://www.cdc.go.kr/CDC/eng/main.jsp (accessed on 2 September 2022)).
